# A normalization strategy applied to HiCEP (an AFLP-based expression profiling) analysis: Toward the strict alignment of valid fragments across electrophoretic patterns

**DOI:** 10.1186/1471-2105-6-43

**Published:** 2005-03-06

**Authors:** Koji Kadota, Ryutaro Fukumura, Joseph J Rodrigue, Ryoko Araki, Masumi Abe

**Affiliations:** 1 Transcriptome Research Center, National Institute of Radiological Sciences (NIRS), 9-1, Anagawa-4-chome, Chiba-shi 263-8555, Japan

## Abstract

**Background:**

Gene expression analysis based on comparison of electrophoretic patterns is strongly dependent on the accuracy of DNA fragment sizing. The current normalization strategy based on molecular weight markers has limited accuracy because marker peaks are often masked by intense peaks nearby. Cumulative errors in fragment lengths cause problems in the alignment of same-length fragments across different electropherograms, especially for small fragments (< 100 bp). For accurate comparison of electrophoretic patterns, further inspection and normalization of electrophoretic data after fragment sizing by conventional strategies is needed.

**Results:**

Here we describe a method for the normalization of a set of time-course electrophoretic data to be compared. The method uses Gaussian curves fitted to the complex peak mixtures in each electropherogram. It searches for target ranges for which patterns are dissimilar to the other patterns (called "dissimilar ranges") and for references (a kind of mean or typical pattern) in the set of resultant approximate patterns. It then constructs the optimal normalized pattern whose correlation coefficient against the reference in the range achieves the highest value among various combinations of candidates. We applied the procedure to time-course electrophoretic data produced by HiCEP, an AFLP-based expression profiling method which can detect a slight expression change in DNA fragments. We obtained dissimilar ranges whose electrophoretic patterns were obviously different from the reference and as expected, most of the fragments in the detected ranges were short (< 100 bp). The normalized electrophoretic patterns also agreed well with reference patterns.

**Conclusion:**

The normalization strategy presented here demonstrates the importance of pre-processing before electrophoretic signal comparison, and we anticipate its usefulness especially for temporal expression analysis by the electrophoretic method.

## Background

Amplified fragment length polymorphism (AFLP) is a DNA fingerprinting technique using electropherograms [1]. AFLP analysis belongs to the category of selective restriction fragment amplification techniques, which are based on the ligation of adapters to genomic restriction fragments followed by PCR-based amplification with adapter-specific primers [2]. This technique has been widely used for genotyping since it requires no prior knowledge of genomic DNA sequences and offers potentially better discriminatory power and speed than the existing techniques for fingerprinting such as random-amplified polymorphism DNA markers (RAPD) [3-8]. However, it has only been used to a limited extent for expression analysis [9]. The main problems with the comparison of AFLP patterns are (i) variation in peak height, and (ii) false positive peaks which often overlap with real peaks, probably due to differences in PCR efficiency [5,10]. There is room for tuning selective PCR amplification [8].

Recently, we developed an AFLP-based gene expression profiling method called HiCEP (High Coverage Expression Profiling) [11]. The experimental and analytical procedures are essentially the same as those of AFLP, i.e., the technique is based on the selective PCR amplification of restriction fragments from a total restriction digest of genomic DNA. Refinements of the selective PCR technique improved reproducibility and reduced the rate of false positive peaks as well as the number of peaks. They also enabled the digestion of purified genomic DNA with two four-nucleotide recognition restriction enzymes, having a higher cutting frequency, such as *Msp*I and *Mse*I. Consequently, the HiCEP method can detect a slight expression change of transcript-derived fragments (TDFs) with high coverage. The estimated 30,000 transcripts expressed in a cell are divided into 256 subgroups (16 MspI-NN primers * 16 NN-MseI primers) containing approximately 120 PCR-amplified TDFs. This number is small enough to be separated by fluorescent capillary electrophoresis using an automated DNA sequencer such as the ABI Prism 310 (Applied Biosystems). We can achieve higher throughput by using several fluorescent dyes at once [14,15].

Normally, digitized electropherograms are imported into image analysis software such as GeneScan (Applied Biosystems), which outputs each fragment (band) together with its length (in bp), area and height (signal intensity), carrying out accurate fragment sizing and background subtraction for most of the operations. GeneScan is capable of separating the signal from each fluorophore to provide higher throughput analysis. However, it should be noted that intense signals from abundant TDFs can breed into each other, potentially confusing the fragment sizing [7,15]. Furthermore, the use of a frequently matching 4-bp cutting endonuclease (*Mse*I) tends to produce many small TDFs (< 100 bp) and in our experience this range is prone to errors of fragment sizing. Cumulative errors of fragment sizing interfere with normalization across different electropherograms and lead to the mis-assignment of valid TDFs. Hence, more detailed analysis such as observation of gradual expression changes in the time series of a TDF still counts in subjective visual examination [11]. Further preprocessing of the electrophoretic data to be compared, each of which is independently normalized according to molecular weight standards, is needed.

The purpose of the present study is to develop a normalization method for the automated analysis of temporal electrophoretic data. We assume the samples to be compared are identical, that TDFs have similar fragment lengths across electropherograms and that expression changes can be detected as variations in peak height using the HiCEP technique. The performance of the method is demonstrated by analyzing a large set of time-course data obtained from mouse embryonic stem (ES) cells, using HiCEP.

## Results and discussion

We analyzed a total of 2560 HiCEP electropherograms (256 sets of ten), containing time-course data of embryonic stem (ES) cells 0, 12, 24, 48, and 96 h after adding stimulation for differentiation. Reproducibility was confirmed by the duplication. We applied the current method to each of the 256 sets.

### Delineation of quality profiles for lanes

**Figure 1:**
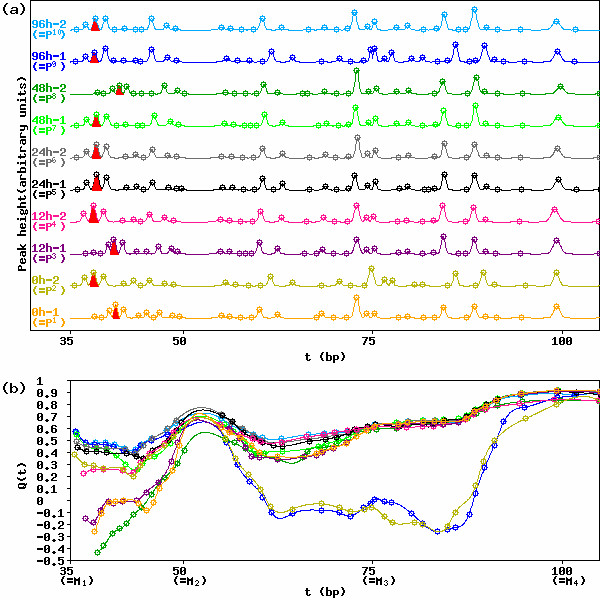
**Electrophoretic patterns and the quality profiles for ten lanes from a primer combination of *CT-tt***. Samples are mouse embryonic stem (ES) cells 0, 12, 24, 48, and 96 h after differentiation. There are ten lanes since each sample are duplicated. From bottom to top: *0 h-1*, *0 h-2*, *12 h-1*, *12 h-2*, *24 h-1*, *24 h-2*, *48 h-1*, *48 h-2*, *96 h-1*, and *96 h-2*. Data from a primer combination of *CT-tt *in the interesting range (35–102 bp) are shown. (a) The approximated electrophoretic lane data and, (b) its interpolated quality profile. An example of calculation of quality profiles for lane *0 h-1 *is shown in Table 1. Note the variation in the lengths of particular TDFs across peaks of lanes (red filled peaks).

When a set of electrophoretic data is arranged and surveyed, one can often find ranges (called 'dissimilar ranges') in which peak fragment lengths are incorrectly measured. For example, in Fig. 1a three lanes (*0 h-1*, *12 h-1*, and *48 h-2*) in the range (35–50 bp) appear to be compressed on the short side. This is probably because another intense peak just under 35 bp is mistaken for the 35 bp marker peak. This reduces the overall similarity between lanes and makes it difficult to recognize identical TDFs such as red filled peaks in Fig. 1a.

To this end, we first developed a method for displaying dissimilar ranges. The method is based on a moving-fragment approach that continuously determines the average correlation coefficient between particular lane *P*^*target *^and the other lanes within a certain range using equation 3. By using the average correlation coefficients, we can make a quality score function *Q*^*k *^(*t*) for all lanes (*k *= 1, 2, ..., 10) at arbitrary length *t *(see Methods). An example of the calculation for lane *0 h-1 *is shown in Table 1. The 'quality profiles' delineated from *Q*(*t*) take the place of detailed visual evaluation of dissimilar ranges (Fig. 1b). Undoubtedly, false peaks must have been used incorrectly at 35 bp in three lanes (*0 h-1*, *12 h-1*, and *48 h-2*) and at 75 bp in two lanes (*0 h-2 *and *96 h-1*).

### Detection of dissimilar ranges

Next, we applied a simple method for the automated detection of dissimilar ranges to 256 sets of electrophoretic data (see Method). The method identified a total of 362 dissimilar ranges. Most (289, 79.8%) of the ranges were of 100 bp or less. This is reasonable because the main source of fragment sizing errors is the presence of intense peaks near the marker [7,11,15] and the HiCEP technique tends to produce short fragments. In fact, of a total of 222,108 detected peaks in the range (35–700 bp) analyzed by GeneScan, 58,988 (26.6%) were < 100 bp.

Visual examination revealed many of those ranges to be genuine, but not all. The set of ten electropherograms shown in Fig. 1 is a good example. Our method identified seven ranges as dissimilar: five lanes (*0 h-1*, *0 h-2*, *12 h-1*, *12 h-2*, and *48 h-2*) in range (35–50 bp) and two lanes (*0 h-2 *and *96 h-1*) in range (50–100 bp). Of these, we at first suspected that two lanes (*0 h-2 *and *12 h-2*) in range (35–50 bp) were false-positives (mistakenly identified as dissimilar). However, we observed that the range in the two lanes is worthy of being normalized: the fragment lengths on the short side of the range deviate gradually from the mean lengths of lanes *24 h-1*, *24 h-2*, *96 h-1*, and *96 h-2 *[see Additional file 1].

Visual examination of all the electropherograms did not reveal any false-negative errors (overlooked dissimilar ranges). Recall that the samples to be compared are identical and that the measure of the quality of fragment sizing is based on a calculation of the average correlation between electropherograms. These results suggest that the normalization strategy we present here is useful, especially for temporal expression analysis.

The effectiveness of the method depends on the choice of the parameter *T *in equation 3 in the Methods section, which is the number of consecutive fragments making up the quality profile examined by the program. Quality profiles using the shortest span (*T *= 1) are noisier than those using a moderate span, and runs using spans of less than four fragments were found unsatisfactory in our investigation. On the other hand, long spans (*T *= 10) tended to miss small dissimilar ranges. These trends are essentially the same as those in the delineation of hydropathy plots of proteins using a moving-window approach and in the detection of transmembrane regions [16]. Although we set *T *= 5 throughout the analysis, further improvement in the choice of parameters as well as the method for the detection of dissimilar ranges remains to be studied.

### Normalization of dissimilar ranges

To normalize dissimilar ranges across a set of electropherograms, it is necessary to select one as a reference. In conventional algorithms the reference is selected manually [17,18]. For reproducible automated normalization, it is vital that the choice be objective. Our method selects the lane (electropherogram) having the highest average quality score in a given dissimilar range. In the case of Fig. 1, our method selects *96 h-2 *as the best reference in ranges (35–50 bp) and (50–100 bp). We cannot, of course, reject the possibility that accurate fragment sizing is performed in the minority group (such as lanes *0 h-1*, *12 h-1*, and *48 h-2 *in range (35–50 bp) in Fig. 1), but it is natural that the best reference should be selected from lanes in the majority group.

**Figure 2:**
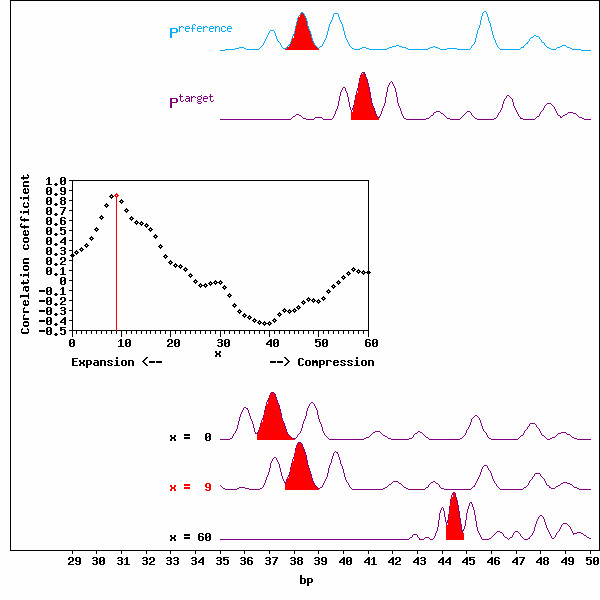
**Normalization for lane *12 h-1 *in dissimilar range (35–50 bp) in Fig. 1 (Model 1)**. Magnified expression profiles of the target *12 h-1 *and the reference *96 h-2 *in the range in Fig. 1 are shown (*top*). Colours are the same as those in Fig. 1. There are 61 possible combinations in this case: 30 different levels of expansion (*x *= 0, 1, ..., 29), the original target profile (*x *= 30), and 30 compressions (*x *= 31, 32, ..., 60). The highest correlation coefficient between the best approximating profile and the reference in range (3–-50 bp) was 0.844 for the case *x *= 9. The position of *x *on the X axis corresponds to the new position of the short side (originally, 35 bp) of the original profile after expansion or compression. For example, the new position of the short side after maximum expansion (*x *= 0) becomes 29 bp, while after maximum compression (*x *= 60) it becomes 41 bp. Visual evaluation of three representative approximate profiles (*x *= 0, 9, and 60) in range (35–50 bp) confirmed the validity of the normalization (*bottom*).

We prepared two models for accurate normalization of various types of fragment sizing errors. Model 1 is the case of an incorrect fragment sizing at the shortest (or longest) marker peak. Figure 2 shows an example of normalization using Model 1. The best approximating profile (normalized profile) is determined by considering various combinations of candidates from *D *× 100% expansion (or - *D *× 100% compression) to *D *× 100% compression of the short side of the original profile at intervals of *d *bp. The best approximating profile is one of the candidate profiles with {*x *× *d *- *D *× (*C*_*e *_- *C*_s_)} / (*C*_*e *_- *C*_s_) × 100% compression of the side in a given range (*C*_*s *_- *C*_*e *_bp), where *x *= {0, 1, ..., 2 × (*C*_*e *_- *C*_s_) × *D */ *d*}. There is of course a trade-off between the computation time and the normalization accuracy in the choices of parameters. In Model 1, we set *D *= 0.4 and *d *= 0.2. We expected that the normalization would be achieved by a linear expansion of the short side of the dissimilar range (35–50 bp) by anchoring the long-side in the target lane *12 h-1*. Indeed, the best approximating profile that achieved the highest correlation coefficient against the reference *96 h-2 *was the case of *x *= 9 (28% expansion).

**Figure 3:**
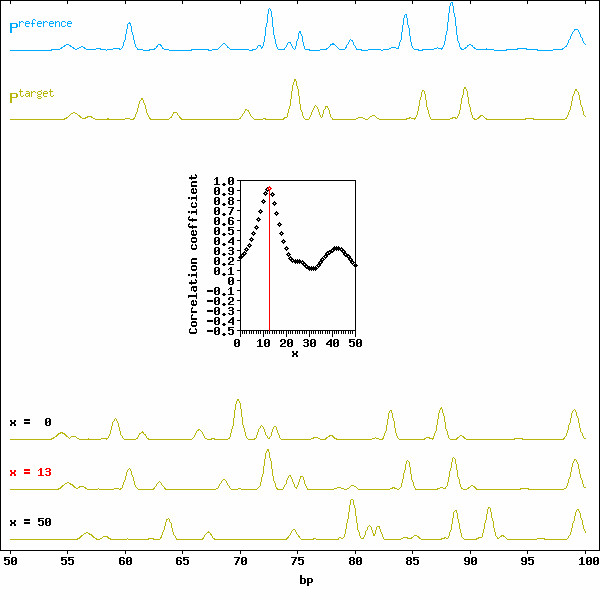
**Normalization for lane *0 h-2 *in the dissimilar range (50–100 bp) in Fig. 1 (Model 2)**. Magnified expression profiles of the target *0 h-2 *and the reference *96 h-2 *in range (50–100 bp) are shown (*top*). Colours are the same as those in Fig. 1. There are 51 possible combinations in this case. The highest correlation coefficient between the best approximating profile and the reference in the range (50–100 bp) was 0.911 for the case *x *= 13. Visual evaluation of three representative approximate profiles (*x *= 0, 13, and 50) in the range confirmed the validity of the normalization (*bottom*).

Figure 3 shows an example of normalization using Model 2. Model 2 is the case of an incorrect fragment sizing at the marker length *M*_*j *_in a dissimilar range (*M*_*j*-1_-*M*_*j*+1 _bp) (see Methods). Accordingly, the program can easily determine the length of 75 bp because there is only one marker length inside of the range (50–100 bp). We can directly apply the normalization procedure for Model 1 to Model 2 by considering two hypothetical dissimilar ranges, (50–75 bp) and (75–100 bp). The main difference from Model 1 is that the two ranges cannot be normalized independently in Model 2: {*x *× *d *- *D *× (100 - 50)}/(100 - 50) × 100% compression (resp. expansion) of the long-side of the original profile in range (50–75 bp) and {*x *× *d *- *D *× (100 - 50)}/(100 - 50) × 100% expansion (resp. compression) of the short side in range (75–100 bp) affect on each other. In Model 2, we set *D *= 0.1 and *d *= 0.2 as a maximal realistic displacement. The best approximating profile is the case of *x *= 13 and is consistent with the reference profile.

**Figure 4:**
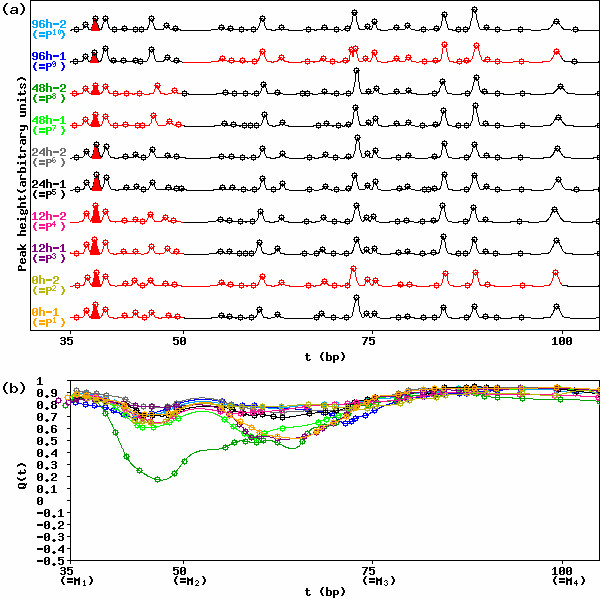
**Electrophoretic patterns and the quality profiles after normalization in Fig. 1**. (a) Normalized electrophoretic patterns. Ranges coloured in red were detected as dissimilar and normalized. Note that the *0 h-2 *consists of two dissimilar ranges: (35–50 bp) and (50–100 bp). After normalization the valid (red filled) peaks are much closer together. (b) Consequently, the more accurate fragment lengths and peak areas in the ranges are accompanied by an increase in the quality scores.

Figure 4 shows the result of normalization for electrophoretic patterns in the primer combination of Fig. 1. Seven dissimilar ranges (coloured in red; five in range (35–50 bp) and two in range (50–100 bp); *0 h-2 *has two normalized ranges) are normalized nearly perfectly (Fig. 4a). For example, the electrophoretic pattern of *0 h-2 *in range (35–50 bp) which is a possible false-positive error are normalized as 2.7% compression of a short side of the range. The correlation coefficients between the target *0 h-2 *and the reference *96 h-2 *in the range before and after normalization are 0.674 and 0.798, respectively.

A quality profile for lane *48 h-2 *indicates that an incorrect normalization is performed in range (35–50 bp) of the lane. The low correlation coefficient (0.4) between the normalized profile and the reference *96 h-2 *in the range, compared to values (> 0.7) between four other normalized profiles (*0 h-1*, *0 h-2*, *12 h-1*, and *12 h-2*) and the reference in the corresponding range, strengthens this suspicion [see Additional file 2]. After visual examination it was decided that the dissimilar range (35–50 bp) of lane *48 h-2 *should be extended on the long side. We searched for the best range to be normalized and chose (35–53.6 bp). The correlation coefficient of the normalized profile, expanded by 26.3% on the short side in the range (35–53.6 bp), was 0.9. Undoubtedly an exhaustive search for edges in dissimilar range might yield better normalization for some cases. However, it also dramatically increases the possible combinations of normalization candidates. It is a balance between the computation time and the number of analyzable TDFs.

One way to do objective evaluation of normalized electrophoretic patterns is to re-delineate the quality profiles (Fig. 4b). Generally, a higher quality score *Q*^*k *^(*t*) for lane *k *indicates greater consistency with the other lanes around arbitrary length *t *if the sample is identical (*e.g*., time-course data). The quality scores after normalization overall were higher than before (Figs. 1b and 4b). This means the assignments of the quality scores to time-course electrophoretic data are effective for evaluating reproducibility.

### Evaluation of the method

The normalization method we propose here can be regarded as an image warping method which deforms images by mapping between image domains [19]. There are a number of reports on warping methods especially for dealing with two-dimensional (2-D) images [19-21]. There are also some methods for 1-D electrophoretic data [17,18,22]. Comparison with these methods might provide an objective evaluation of the current method. However, they are not directly comparable with the current method because of different frameworks such as input data format, the requirement of pre-determined parameters, and so on [17,22].

A critical step in the analysis of 1-D electrophoretic data is the assignment of the correct size to each TDF. In time-course data, one expects that the same TDFs should have quite close fragment lengths across electropherograms and that temporal expression changes are reflected as differences in peak height. We developed the current method aimed at temporal expression analysis by the electrophoretic method and used a scoring system for an objective evaluation of experimental reproducibility using *Q*^*k *^(*t*) which indicates a relative similarity at *t *(bp) in lane *k *to the other lanes. We demonstrate two other sets of electrophoretic data and discuss the feasibility of the method.

**Figure 5:**
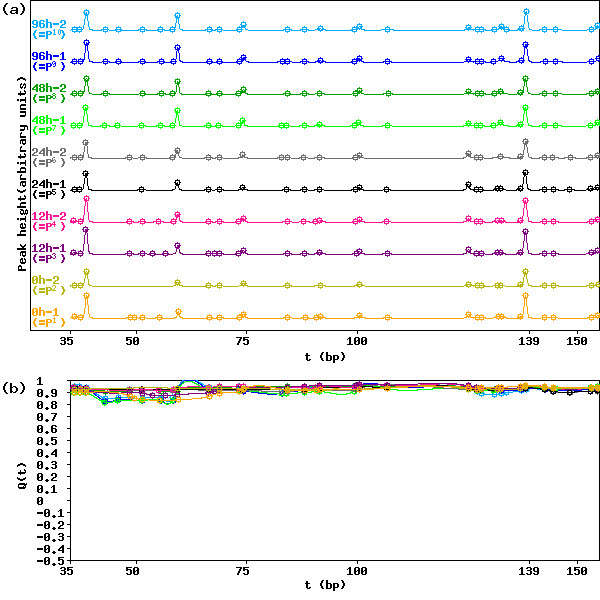
**Reproducible electrophoretic patterns and the quality profiles for ten lanes**. Data from a primer combination of *AA-gc *in the interesting range (35–155 bp) are shown. (a) The electrophoretic data lane and (b) its quality profile.

Figure 5 shows a set of electrophoretic patterns and quality scores which is different from the primer combination used in Figs. 1, 2, 3, 4. This is a representative example of electrophoretic patterns with high quality scores (arbitrary defined as > 0.7). Visual evaluation confirmed the reproducibility of the set of ten electrophoretic patterns throughout the analyzed range (35–700 bp). There is, of course, no dissimilar range detected by the current method.

**Figure 6:**
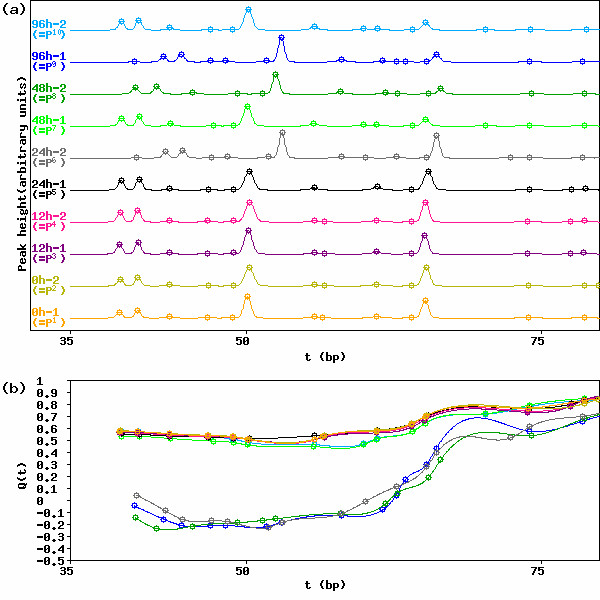
**Electrophoretic patterns and the quality profiles for ten lanes from a primer combination of *GA-gc***. Data from a primer combination of *GA-gc *in the interesting range (35–80 bp) are shown. (a) The electrophoretic data lane and (b) its quality profile. Three lanes (*24 h-2*, *48 h-2*, and *96 h-1*) have a dissimilar range (35–75 bp) suitable for both normalization Models 1 and 2.

**Figure 7:**
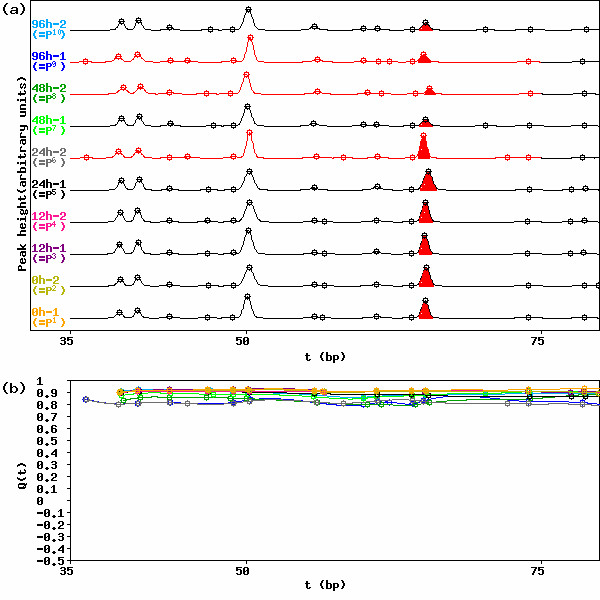
**Electrophoretic patterns and the quality profiles after normalization in Fig. 6**. (a) Normalized electrophoretic patterns. Ranges coloured in red were detected as dissimilar and normalized. After normalization the valid (red filled) peaks are much closer together.

We should demonstrate the case of normalization to dissimilar range (35–75 bp) where both Models 1 and 2 are applicable. A set of ten electrophoretic patterns and their quality scores shown in Figure 6 is the good example. There are three lanes with dissimilar range (*24 h-2*, *48 h-2*, and *96 h-1*) detected by the method. Of these, *24 h-2 *and *96 h-1 *were normalized using Model 1 and *48 h-2 *was normalized using Model 2. Visual evaluation of the electrophoretic patterns and the quality scores after normalization verified the choices of the models as appropriate (Figure 7). The use of normalized electrophoretic patterns facilitates the identification of TDFs (e.g., red filled fragments in Fig. 7) having potential temporal expression change. The development of a peak alignment algorithm for multiple lanes and integration with the current method are the next challenge.

We also estimated the feasibility of the method with regard to an increasing number of peaks with certain quality score or more. The minimum value of *Q*(*t*) necessary for the accurate alignment of valid TDFs across lanes is about 0.7 (Fig. 4b). Accordingly, we set the threshold to be 0.7. The number of peaks with *Q*(*t*) ≥ 0.7 in the range (35–700 bp) before and after normalization are 202,204 (91.0% of the total number of peaks in the range detected by GeneScan) and 205,829 (92.7%), respectively. Furthermore, 3,334 (92%) of the 3,625 (= 205,829 - 202,204) new high-quality peaks were < 100 bp, which corresponds to the biased distribution of the detected dissimilar ranges (nearly 80% of which were 100 bp or less).

## Conclusion

When we apply the method to HiCEP time-course data, we assume that the set of electrophoretic data to be compared is identical (*i.e*., corresponding TDFs across electropherograms should have nearly the same fragment lengths). The monitoring of temporal expression change by the HiCEP technique has great potential for screening of genes related to chemotherapeutic drug resistance, circadian rhythm, and so on [11,23,24]. Although the current method was developed for pre-processing HiCEP data, the algorithm is easily applicable to the processing of other 1-D electrophoretic data such as AFLP and DD if the samples are identical or nearly identical. We strongly recommend the strategy be widely used for data processing for temporal expression analysis by the electrophoretic method.

## Methods

### Samples

mRNAs were prepared from mouse embryonic stem (ES) cells at 0, 12, 24, 48, and 96 h after removal of Leukemia Inhibitory Factor (LIF) from the culture medium. The samples subjected to HiCEP reaction were duplicated. We designated each sample as *0 h-1*, *0 h-2*, *12 h-1*, *12 h-2*, *24 h-1*, *24 h-2*, *48 h-1*, *48 h-2*, *96 h-1*, and *96 h-2*.

### HiCEP analysis

mRNAs prepared from each sample were digested with two 4-bp-cutting endonucleases (*Msp*I combined with *Mse*I) and ligated with the corresponding adaptors. The resulting HiCEP templates, MspI-MseI-poly(A) mRNAs, were amplified by fluorescently labelled primers; for labelling, FAM, HEX, and NED were used. In total, 256 primer combinations (16 MspI-NN primers combined with 16 NN-MseI primers; N = {A, C, G, T}) were used in the HiCEP analysis. For example, a primer combination of MspI-TA and GC-MseI is capable of amplifying particular transcript-derived fragments (TDFs) corresponding to that combination. The details of the protocol of the HiCEP reaction are described elsewhere [11]. An animation of the principle is provided at the following URL .

### Electrophoresis and image analysis

The PCR products were denatured and loaded on an ABI Prism 310 (Applied Biosystems) for capillary gel electrophoresis. The digitized images were analyzed by the GeneScan software (Applied Biotech). The size of the fragments was calculated by the software, according to internal molecular size markers of 35, 50, 75, 100, 139, 150, 160, 200, 300, 340, 350, 400, 490, 500, 600, and 700 bp, on each gel. The fragment sizing and baseline subtraction were performed by the software. The software quantifies each peak by the fragment length *L *(in bp), peak height *H*, and area *A *(in arbitrary units). Accordingly, the subsequent normalization procedure accepts these three-tuples as input for detected TDFs between 35 bp and 700 bp. TDFs smaller than 35 bp or larger than 700 bp were omitted from the analysis because the range was outside the size calibration range.

### Delineation of quality scores for lanes

The starting point of normalization is a set of lanes (10 time-course measurements; 0, 12, 24, 48, and 96 h, each experiment duplicated) in each of 256 primer combinations. We explain the procedure using data from the primer combination of '*Msp*I-CT combined with tt-*Mse*I (designated as *CT-tt*)' because the ten electropherograms have some ranges for which fragment sizing is obviously inappropriate (we therefore designated such ranges as "dissimilar ranges").

The first step starts from the Gaussian approximation of each lane. The use of the approximating lane is the same as described in Aittokallio et al. [25-27]. Briefly, a fragment *F*_*i *_in lane *P *is originally characterized by the three-tuples (*L*_*i*_, *H*_*i*_, *A*_*i*_). If lane *P *consists of *n *fragments 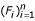, the approximation of the lane at length *t *is given by:


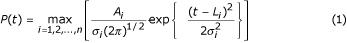


where *σ*_*i *_is obtained from the following equation:


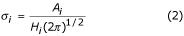


The approximation is performed independently for each lane. The ten approximate profiles of time-course data in the primer combination of *CT-tt *are shown in Fig. 1a.

For the automated identification of 'dissimilar ranges' from the expression profiles of ten lanes 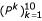, we next assign quality scores to each of the fragments 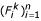, where the fragments are originally numbered with respect to their lengths. By using the ten approximate profiles, relative similarity scores 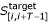 for intervals from fragment *i *to fragment (*i*+*T*-1) (*i *= 1, 2,..., *n *- *T *+ 1) in lane *P*^*target *^(*target *= {1, ..., 10}) are calculated from the following equation:


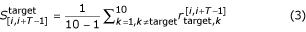


where 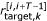 is the Pearson correlation coefficient between the target lane *P*^*target *^and one of the other lanes *P*^*k *^in the interval (*start*-*end *bp) which always includes *T *fragments from fragment *i *to fragment (*i*+*T*-1) (*i *= 1, 2, ..., *n*-*T*+1). The interval is defined as: *start *= *L*_*i *_- 2.5*σ*_*i *_and *end *= *L*_*i*+*T*-1 _+ 2.5*σ*_*i*+*T*-1_. In this analysis, the number of fragments *T *is held constant at *T *= 5; other numbers are of course possible. By applying a moving window of *T *fragments, most of the fragments (*n*-*T*+2 fragments in this case, with the exception of *F*_1_, *F*_2_, *F*_3_, *F*_4_, *F*_*n*-3_, *F*_*n*-2_, *F*_*n*-1_, and *F*_*n*_) have *T *relative similarity scores. Finally, the relative quality value *Q*(*L*_*i*_) for fragment *F*_*i *_is defined as the average of the similarity scores which satisfy *start *≤ *L*_*i *_≤ *end*. An example of the calculation is given in Table 1. Quality scores at arbitrary lengths *t*, *Q*(*t*), are interpolated by the use of cubic splines to 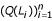. The procedure is applied to each of the ten lanes 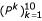 and then the quality profiles 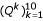 corresponding to the expression profiles are created (Fig. 1b).

The quality profiles delineated from *Q*(*t*) have a clear interpretation. The high (or low) score for *Q*^*k*^(*t*) in lane *k *indicates a high (or low) level of relative similarity between the lane and the others around the length *t*.

### Detection of dissimilar ranges

Now we have information (quality profiles) for the automated detection of dissimilar ranges. Here we adopt a simple method for detecting the range. Briefly,

1) Seek 'seed' ranges (*C*_*seed_s *_- *C*_*seed_e *_bp) which satisfy two conditions: a) *Q*(*t*) ≤ *thres*_*seed *_and b) they contain at least two peaks.

2) Seek *C*_*tmp_s *_which satisfies both 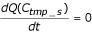 and *C*_*tmp_s *_<*C*_*seed_s*_; similarly, *C*_*tmp_e*_, 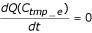 and *C*_*tmp_e *_<*C*_*seed_e*_

3) Substitute the nearest marker length 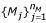 (in this case, *M*_1 _= 35, *M*_2 _= 50, ..., 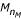 = 700) to *C*_*tmp_s *_(resp. *C*_*tmp_e*_) for *C*_*s *_(resp. *C*_*e*_); accordingly, both *C*_*s *_and *C*_*e *_= 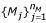 and *C*_*s *_<*C*_*e*_

Aparameter *thres*_*seed *_is set to 0.3 empirically. Forexample, *P*^9 ^has the following parameters in Fig. 1b: *C*_*seed_s *_= 57.04, *C*_*seed_e *_= 89.98, *C*_*tmp_s *_= 52.60, *C*_*tmp_e *_= 104.60, *C*_*s *_= *M*_2_, and *C*_*e *_= *M*_4_. Although fine tuning might be necessary, the procedure enables us to display dissimilar ranges.

### Selection of the reference lane

When we want to correct a dissimilar range (*C*_*s *_- *C*_*e *_bp), we have to select the "reference" (a kind of mean or typical profile in the corresponding range). One method is to choose lane *P*^*reference *^satisfying max {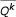}, where 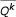 is the average of *Q*^*k *^in the range (*C*_*s *_- *C*_*e *_bp) in lane 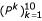. For example, the algorithm selects *P*^10 ^(i.e., *96 h-2*) as a reference in a particular range (*M*_1_-*M*_2 _bp) and also in range (*M*_2_-*M*_4 _bp).

### Two models for the normalization of dissimilar ranges

The meaning of the word "normalization" here is to correct the fragment lengths (*L*) and the areas (*A*) of peaks in a dissimilar range so that the similarity between the normalized electrophoretic pattern and the reference pattern in the corresponding range can be maximized. To normalize a particular lane *P*^*target *^against the reference *P*^*reference*^, we now consider the following two models. Model 1 is the case of an incorrect fragment sizing at the shortest (or longest) marker peak, i.e, *C*_*s *_= *M*_1 _= 35 (or *C*_*e *_= 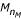 = 700). The peak lengths deviate more and more from the reference length moving from *C*_*e *_to *C*_*s *_(or from *C*_*s *_to *C*_*e*_). Model 2 is the case of an incorrect fragment sizing near marker length *M*_*j *_(*C*_*s *_<*M*_*j *_<*C*_*e*_, *j *= {*2*, *3*,..., *n*_*M *_- 1}; the inside of dissimilar range (*C*_*s *_- *C*_*e *_bp)). Roughly, the deviation of peak lengths from the reference length gradually increases starting from *C*_*s*_; the maximum deviation is reached at *M*_*j *_(*C*_*s *_<*M*_*j *_<*C*_*e*_); the deviation decreases gradually; and finally disappears at *C*_*e *_bp.

Normalization is performed by either expanding or compressing. Consider, for example in Model 1, normalization for the expression profile of *P*^3 ^(*12 h-1*) in range (*M*_1_-*M*_2 _bp) against the reference *P*^10 ^(*96 h-2*). Undoubtedly, the profile displays a systematic deviation from the reference. The degree of the deviation gradually increases starting from *M*_2 _bp to *M*_1 _bp probably because an intense peak generated near the shortest marker peak for the correction of *M*_1 _bp is used mistakenly. We expect the normalization will be achieved by a linear expansion of the short side (*M*_1_) of the range (*M*_1_-*M*_2 _bp) by anchoring the long side. The best approximating profile is found by considering various combinations of normalization candidates starting from *D *× 100% expansion to *D *× 100% compression of the short side at intervals of *d *bp. We set *D *= 0.4, as a maximal realistic displacement and *d *= 0.2. Accordingly, in practice, the number of combinations is 2 × (*C*_*e *_- *C*_*s*_) × *D*/*d *+ 1 (for example, there are 61 combinations of normalization candidates in the range (*M*_1_-*M*_2 _bp)) in Model 1.

For each combination *x *(*x *= {0, 1, ..., 2 × (*C*_*e *_- *C*_*s*_) × *D*/*d*)}, we make a candidate profile *P*_*x *_by changing three parameters (*L*_*i*_, *A*_*i*_, and *σ*_*i*_) accompanied by fragments (*F*_*i*_) in the dissimilar range (*C*_*s *_- *C*_*e *_bp), according to the level of correction (expansion or compression). Those parameters are calculated as follows:


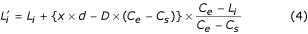



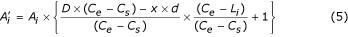



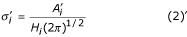


Candidates are made by substituting these transformed three-tuples 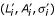 in a given range (*C*_*s *_- *C*_*e *_bp) into eq. (1). The best approximate profile is the one that achieves the highest correlation coefficient between *P*^*reference *^and *P*_*x *_(*x *= {0, 1, ..., 2 × (*C*_*e *_- *C*_*s*_) × *D */ *d*}) in the range (*C*_*s *_- *C*_*e *_bp). In the normalization for the expression profile *P*^3 ^in the range (*M*_1_-*M*_2 _bp) against the reference *P*^10^, the best normalized profile by our method matches well with the reference (Fig. 2).

A good example of Model 2 is the expression profile *P*^2 ^(*0 h-2*) in range (*M*_2_-*M*_4 _bp) with the reference *P*^10 ^(*96 h-2*); there is no possibility of Model 1 (*C*_*s *_≠ *M*_1_) and the number of incorrect marker lengths is only one (*M*_3_). Model 2 is a mixture of Model 1. The normalization is also done by one of the {2 × (*C*_*e *_- *C*_*s*_) × *D */ *d *+ 1} combinations starting from *D *× 100% compression of the long side in (*M*_2_-*M*_3 _bp) and *D *× 100% expansion of the short side in (*M*_3_-*M*_4 _bp) to *D *× 100% expansion of the long side in (*M*_2_-*M*_3 _bp) and *D *× 100% compression of the short side in (*M*_3_-*M*_4 _bp) at intervals of *d *(= 0.2) bp. Unlike Model 1, we set *D *= 0.1 as a maximal realistic displacement. In the normalization for the expression profile *P*^2 ^in the range (*M*_2_-*M*_4 _bp) against the reference, the best normalized profile by our method is matches well with the reference (Fig. 3).

It should be noted that when a dissimilar range (*M*_*j*_-*M*_*j*+*l *_bp) is very wide (*j *= 1, 2,..., *n*_*M *_- *l*; *l *≥ 3), there are two or more possibilities for incorrect marker lengths in Model 2. Of these cases, we only consider cases with *j *= 1 in Model 1 because such cases are the only realistic ones. For the remaining cases (*j *= 2,..., *n*_*M *_- *l*; *l *≥ 3), the experiment should be redone rather than trying to normalize them by considering numerous possibilities. It should also be noted that there is a case of a dissimilar range (*M*_1_-*M*_3 _bp) to which both Models 1 and 2 are applicable. In this case, the best approximate profile is decided by comparing the two best possible profiles determined using Models 1 and 2.

## Authors' contributions

KK invented the method and wrote the paper. RF made critical comments in light of the HiCEP experimental technique. JJR edited the paper. RA and MA provided critical comments and led the project.

## Supplementary Material

Additional File 1 **Magnified electrophoretic patterns and the quality profiles in range (35–50 bp) in **Fig. 1. Descriptions are the same as those in Fig. 1. Detailed observation of the dissimilar range for two lanes (*0 h-2 *and *12 h-2*) confirmed the identification. Click here for file

Additional File 2 **Magnified electrophoretic patterns and the quality profiles in range (35–50 bp) in **Fig. 4. Descriptions are the same as those in Fig. 4. Visual evaluation confirmed the validity of the normalizations (2.7% compression of the short side of the range) for two lanes (*0 h-2 *and *12 h-2*) which are suspected false-positive errors. Click here for file

## Figures and Tables

**Table 1:** Calculation of quality scores for lane *0 h-1 *in Fig. 1. Similarity scores (*S*) are computed using Equation 3. The quality score at fragment length *L*_4 _is calculated as (0.00 + 0.17 + 0.04 - 0.27)/4 = -0.02. The quality profile for each lane is made by spline interpolation of a set of quality scores of fragment lengths of peaks in the lane.

*i*	*L*_*i*_	*σ*_*i*_	*S*^1 ^in size interval [*L*_*i*-*j *_- 2.5 *σ *_*i*-*j*_, *L*_*i*-*j*+4 _+ 2.5 *σ*_*i*-*j*+4_]					*Q*(*L*_*i*_)
				
			*j *= 0	*j *= 1	*j *= 2	*j *= 3	*j *= 4	
1	38.28	0.145	-0.27	-	-	-	-	-0.27
2	39.16	0.143	0.04	-0.27	-	-	-	-0.11
3	40.17	0.186	0.17	0.04	-0.27	-	-	-0.02
4	41.06	0.212	0.00	0.17	0.04	-0.27	-	-0.02
5	42.07	0.223	0.02	0.00	0.17	0.04	-0.27	-0.01
6	44.05	0.221	-0.36	0.02	0.00	0.17	0.04	-0.03
7	45.13	0.190	-0.28	-0.36	0.02	0.00	0.17	-0.09
8	46.05	0.149	0.52	-0.28	-0.36	0.02	0.00	-0.02
9	46.80	0.215	0.59	0.52	-0.28	-0.36	0.02	0.10
10	48.48	0.240	0.74	0.59	0.52	-0.28	-0.36	0.24
11	49.41	0.242	0.69	0.74	0.59	0.52	-0.28	0.45
12	54.95	0.320	0.49	0.69	0.74	0.59	0.52	0.61
13	56.09	0.215	0.30	0.49	0.69	0.74	0.59	0.56
14	57.54	0.152	0.39	0.30	0.49	0.69	0.74	0.52
15	59.00	0.195	0.41	0.39	0.30	0.49	0.69	0.45
16	60.05	0.252	0.39	0.41	0.39	0.30	0.49	0.40
17	62.41	0.222	0.32	0.39	0.41	0.39	0.30	0.36
18	67.55	0.135	0.43	0.32	0.39	0.41	0.39	0.39
19	68.26	0.259	0.47	0.43	0.32	0.39	0.41	0.40
20	69.76	0.216	0.57	0.47	0.43	0.32	0.39	0.43
