# Supplementary figures and images for: A normalization strategy applied to HiCEP (an AFLP-based expression profiling) analysis: Toward the strict alignment of valid fragments across electrophoretic patterns
Source: BMC Bioinformatics. 2005 Mar 6;6:43. doi: 10.1186/1471-2105-6-43 (PMC554994; doi:10.1186/1471-2105-6-43)

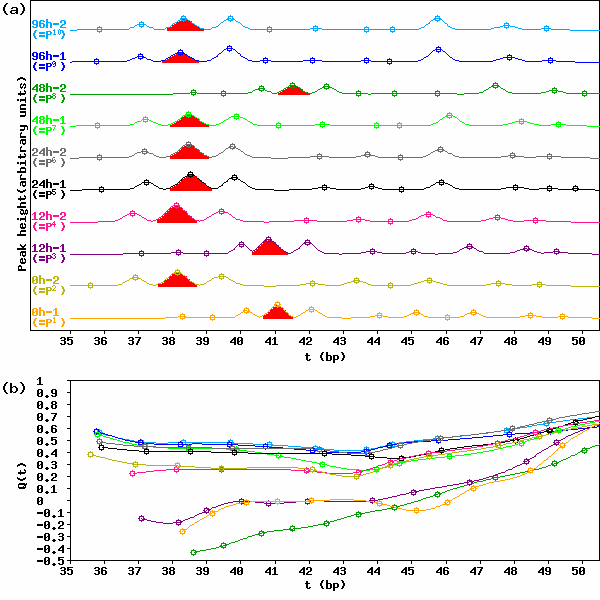

Supplement: Additional File 1 — Magnified electrophoretic patterns and the quality profiles in range (35–50 bp) in Fig. 1. Descriptions are the same as those in Fig. 1. Detailed observation of the dissimilar range for two lanes (0 h-2 and 12 h-2) confirmed the identification. [file 1471-2105-6-43-S1.png]

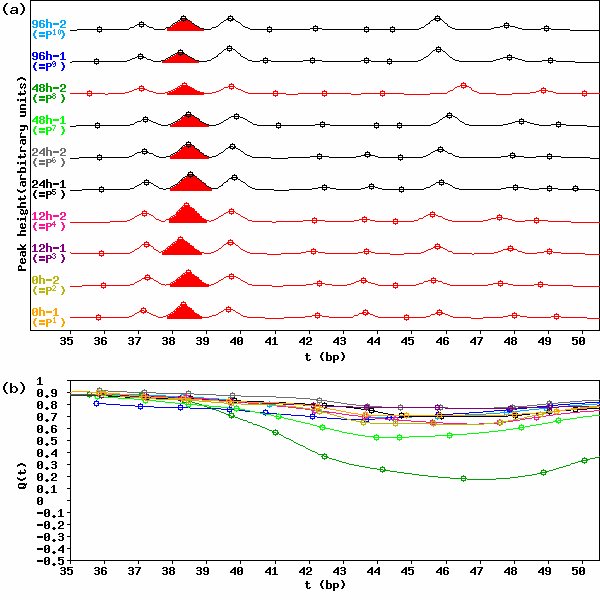

Supplement: Additional File 2 — Magnified electrophoretic patterns and the quality profiles in range (35–50 bp) in Fig. 4. Descriptions are the same as those in Fig. 4. Visual evaluation confirmed the validity of the normalizations (2.7% compression of the short side of the range) for two lanes (0 h-2 and 12 h-2) which are suspected false-positive errors. [file 1471-2105-6-43-S2.png]
